# Suspension of the N. Y. College of Dentistry

**Published:** 1869-06

**Authors:** 


### SUSPENSION OF THE N. Y. COLLEGE OF DENTISTRY.

The difficulties that have for some time existed between the Faculty and Trustees of this institution have resulted in its dissolution. This will be regretted by every true lover of our profession in the country. We hoped in the beginning that the New York College of Dentistry would increase in favor, prestige and efficiency till it should fully meet the wants of the profession in an educational point of view. New York is the metropolis of the country, and on account of its situation and advantages will continue to hold that position. There is gathered there a larger number of the strong men of the profession than at any other point; and, indeed, all the facilities for constituting a large and efficient institution certainly exist in New York. Out of the ashes of this we hope another may spring that shall be free from the disabilities that have proved so fatal; one that shall receive the sustaining care and strong support of the profession of that vicinity.

In reference to this subject we give place to the following from the *American Journal of Dental Science:*

"We regret to learn that the New York College of Dentistry no longer exists, its charter having been annulled by the Supreme Court of the State in an action brought by the Attorney General. This action was instituted on a presentation of the condition of the college by a member of the Faculty, who considered the acts of a majority of its Board of Trustees to be in direct violation of its charter.

"The case came up before Judge Cardozo, and an injunction was granted and a receiver appointed to take charge of the property of the College on the 22d of April last.

"Previous to any steps being taken to annul its charter, no less than six of the most prominent members of its Board of Trustees resigned.

As we are well acquainted with the untiring efforts of its former faculty to make it worthy the support of the profession, and of the pride they felt in its welfare, we deplore its fate and deeply sympathize with those who have expended so much time, labor and means in its behalf.

This is the second College of Dentistry which has been organized in the State of New York, and we had hoped that, unlike its predecessor, its career would be a long and prosperous one.

